# Unlocking microbial potential: advances in omics and bioinformatics for aromatic hydrocarbon degradation

**DOI:** 10.1007/s11274-025-04592-4

**Published:** 2025-10-14

**Authors:** Isamar-Maydeth Vidal-Silva, Antonio Loza, Rosa-Maria Gutierrez-Rios

**Affiliations:** Departamento de Microbiología Molecular, Instituto de Biotecnología, Universidad Nacional Autónoma de México, Avenida Universidad 2001, Colonia Chamilpa, Cuernavaca, Morelos 62210 México

**Keywords:** Monocyclic aromatic hydrocarbons, Polycyclic aromatic hydrocarbons, Microbial degradation, Aerobic and anaerobic, Bioinformatic tools, Genomics and metagenomics

## Abstract

Aromatic hydrocarbons (AHs) are persistent environmental pollutants with high toxicity. Bacterial degradation of AHs provides a sustainable and cost-effective approach for the remediation of sites contaminated with both mono- and polycyclic aromatic hydrocarbons. Aerobic degradation of AHs typically involves oxygenases-mediated hydroxylation followed by aromatic ring cleavage. In contrast, anaerobic degradation relies on diverse activation mechanisms that ultimately converge on the central intermediate benzoyl-CoA. Over the past decades, research on bacterial degradation of AHs has grown steadily, supported by advances in omics and bioinformatics. In this review, we summarize the current knowledge on the pathways, enzymes, and microbial diversity involved in AH degradation, highlighting how omics and bioinformatic approaches are advancing our understanding of this process. However, to improve our knowledge of microbial AHs catabolism, it is crucial to prioritize the characterization of novel enzymes and pathways, especially those mediating anaerobic and hybrid degradation strategies. Addressing this gap requires the development of specialized resources that incorporate a broader taxonomic diversity and an expanded inventory of anaerobic genes and enzymes supported by experimental evidence. Equally important is the integration of multi-omics technologies, artificial intelligence, and ecological modeling into unified analytical pipelines. These efforts will be key to fully unlocking microbial metabolic potential and guiding more effective bioremediation and monitoring strategies for AHs.

## Introduction

Aromatic hydrocarbons (AHs) are organic compounds with at least one benzene ring as a central structural unit. They are broadly divided into monocyclic aromatic hydrocarbons (MAHs) and polycyclic aromatic hydrocarbons (PAHs). MAHs contain a single benzene ring, making them the simplest form of aromatic compounds (Shrivastava and Phale 2013; Ladino-Orjuela et al. 2016). This category includes benzene, toluene, ethylbenzene, and xylene, collectively referred to as BTEX (Shrivastava and Phale 2013; Phale et al. 2020). In contrast, PAHs consist of two or more fused aromatic rings without any heteroatoms. PAHs containing up to three rings are classified as low molecular weight (LMW) PAHs, while those with four or more rings are termed high molecular weight (HMW) PAHs. Over 100 PAHs and their derivatives have been identified, including LMW compounds such as naphthalene, fluorene, anthracene, and phenanthrene, and HMW representatives such as fluoranthene, pyrene, benzo[a]anthracene, and chrysene (Seo et al. 2009; Phale et al. 2020).

AHs are of significant concern due to their toxicity and ubiquity in all environments. Many BTEX and PAHs, along with their metabolized forms, are widely recognized as potent carcinogenic, mutagenic, and teratogenic agents. The carcinogenic properties of AHs are primarily related to their ability to form DNA adducts, which contribute to key gene mutations (Martins et al. 2013). In addition, acute exposure to AHs has been associated with oxidative stress, reproductive disorders, immunosuppression, and endocrine disruption, further complicating their adverse health effects (Cao et al. 2022).

AHs are recalcitrant compounds due to the high thermodynamic stability of the benzene ring. Nonetheless, certain bacteria have evolved specialized metabolic pathways that enable them to utilize AHs as sole sources of carbon and energy (Shrivastava and Phale 2013). Previous studies have summarized core biochemical, genetic, and genomic aspects of bacterial catabolism of AHs (Carmona et al. 2009; Fuchs et al. 2011; Phale et al. 2020; Pandolfo et al. 2023). Since then, research in this field has benefited from rapid advances in sequencing technologies, the expansion of databases, and the development of sophisticated bioinformatic tools. In this review, we summarize the current knowledge on the mechanisms of AH biodegradation and emphasize the role of bioinformatics and omics approaches in revealing novel pathways, enzymes, and microbial diversity, while also highlighting areas that require further research.

## Microbial degradation of aromatic hydrocarbons

Bacterial catabolism of AHs, under both anaerobic and aerobic conditions, is organized into an upper (or *peripheral*) and lower (or *ring-cleavage*) pathway. In the upper pathway, diverse aromatic compounds are funneled into central intermediates such as catechol, protocatechuate, or benzoyl-CoA. In the lower pathway, these intermediates are dearomatized and cleaved, resulting in metabolites that can be used by bacteria in the production of biomass.

### Aerobic catabolic pathways for the degradation of MAHs

A wide range of bacteria catalyze the biodegradation of AHs under oxic conditions, primarily including Gram-negative genera such as *Pseudomonas*,* Sphingomonas*,* Acinetobacter*, and *Ralstonia*, as well as Gram-positive genera like *Rhodococcus*,* Nocardioides*, and *Mycobacterium* (Phale et al. 2020). This process is mediated by oxygenases (monooxygenases and dioxygenases), which catalyze the incorporation of hydroxyl groups (-OH) into the aromatic ring using molecular oxygen (O₂) as a co-substrate. Monooxygenases incorporate one oxygen atom into the substrate while reducing the second to water. In contrast, dioxygenases insert both oxygen atoms into the aromatic ring, resulting in dihydroxylation or ring cleavage (Ladino-Orjuela et al. 2016).

**Fig. 1:**
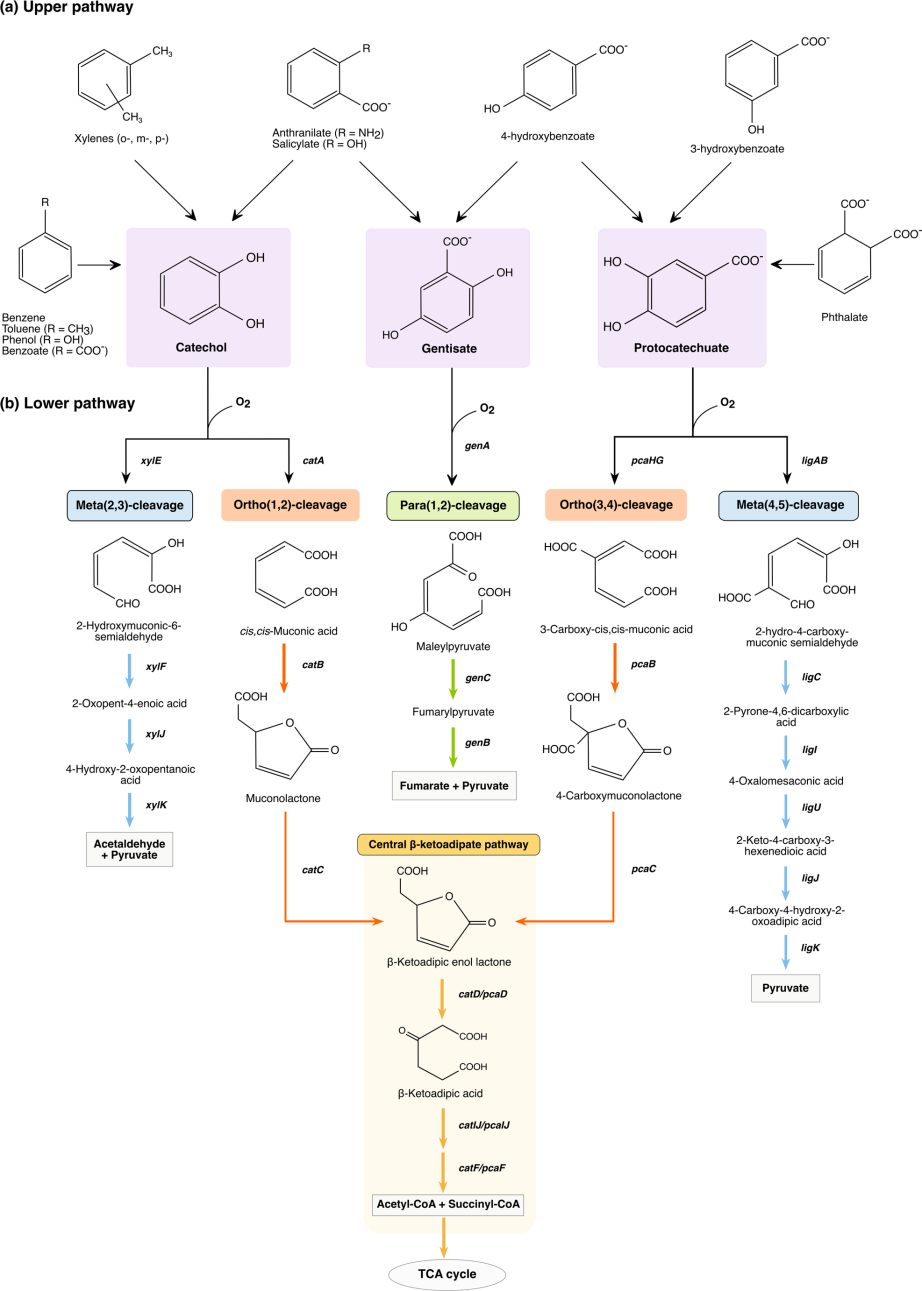
Aerobic catabolism of monoaromatic hydrocarbons. The process follows two main stages: in the upper (peripheral) pathways **(a)**, monoaromatic compounds are activated by monooxygenases or ring-hydroxylating dioxygenases to yield central hydroxylated intermediates (purple boxes). These compounds then enter the lower (central) pathways **(b)**, where ring-cleaving dioxygenases catalyze ortho-, meta-, or para-cleavage. The resulting ring-fission products (light-gray boxes), such as succinate, pyruvate, or acetyl-CoA precursors, are metabolized through the TCA cycle. The ortho-cleavage of catechol and protocatechuate converges into the common β-ketoadipic acid intermediate (β-ketoadipate central pathway). The cat cluster encodes enzymes for catechol ortho(1,2)-cleavage, including catechol 1,2-dioxygenase (*catA*), muconate cycloisomerase (*catB*), and muconolactone isomerase (*catC*). The *pca* cluster of the Protocatechuate ortho(3,4)-cleavage encodes a protocatechuate 3,4-dioxygenase (*pcaHG*), β-carboxy-cis, cis-muconate cycloisomerase (*pcaB*), and a γ-carboxy-muconolactone decarboxylase (*pcaC*). Downstream steps of β-ketoadipate pathway involve β-ketoadipate enol-lactonase (*catD/pcaD*), β-ketoadipate succinyl-CoA transferase (*catIJ/pcaIJ*) and β-ketoadipyl-CoA thiolase (*catF/pcaF*). In contrast, the *xyl* cluster directs the meta(2,3)-cleavage of Catechol through catechol 2,3-dioxygenase (*xylE*), 2-hydroxymuconic semialdehyde hydrolase (*xylF*), 2-hydroxymuconic semialdehyde dehydrogenase (*xylG*), 2-oxopent-4-enoate hydratase (*xylJ*), and a 4-hydroxy-2-oxalovalerate aldolase (*xylK*). Genes involved in the Protocatechuate meta(4,5)-cleavage encode a protocatechuate 4,5-dioxygenase (*ligAB*), 4-carboxy-2-hydroxymuconate-6-semialdehyde dehydrogenase (*ligC*), 2-pyrone-4,6-dicarboxylate hydrolase (*ligI*), 4-oxalomesaconate delta isomerase (*ligJ*), and 4-hydroxy-4-methyl-2-oxoglutarate aldolase (*ligK*). The cluster responsible for the Gentisate para(1,2)‑cleavage pathway comprises genes encoding gentisate 1,2‑dioxygenase (genA), maleylpyruvate isomerase (*genC*), and fumarylpyruvate hydrolase (*genB*)

The upper pathway begins with the activation of aromatic substrate, typically catalyzed by multicomponent ring-hydroxylating dioxygenases (RHDs) that produce cis-dihydrodiol derivatives (Fig. 1). These are subsequently oxidized to central intermediates such as hydroquinone, resorcinol, and catechol, the latter being the most common. Some pathways proceed via non-catecholic intermediates such as protocatechuate, gentisate, salicylate, and homoprotocatechuate (Ladino-Orjuela et al. 2016; Ghosal et al. 2016; Durante-Rodríguez et al. 2018). These hydrolated intermediates are then subjected to an ortho- or meta-cleavage via ring-cleaving dioxygenases (RCDs) in the lower pathway. Ortho-cleavage occurs between two hydroxyl groups and is catalyzed by intradiol dioxygenases. In contrast, meta-cleavage targets the adjacent C–C bond and is mediated by extradiol dioxygenases (Vaillancourt et al. 2006; Phale et al. 2020).

While meta-cleavage pathways exhibit considerable diversity, ortho-cleavage is primarily restricted to the central β-ketoadipate pathway (β-KAP). This pathway features separate branches for the catabolism of catechol and protocatechuate, both converging at β-ketoadipate enol-lactone (Wells and Ragauskas 2012). β-KAP is mostly chromosomally encoded (e.g., *pca* and *cat* genes in *P. putida*), but some exceptions exist, such as the plasmids pWW174 from *Acinetobacter calcoaceticus* and pSymB from *Sinorhizobium melilotii* (Shrivastava and Phale 2013). The pathway is broadly conserved across bacteria, yet its regulation and gene organization show notable diversification (Wells and Ragauskas 2012). For example, gene order within metabolic operons varies across bacterial species, except in cases where genes may coevolve due to their products being subunits of a single enzyme and are co-expressed. An example of this is the consecutive genes *pcaHG* and *pcaIJ*, which encode subunits of protocatechuate 3,4-dioxygenase and 3-oxoadipate CoA-transferase, respectively (Buchan et al. 2004) (Fig. 1).

Recent research has increasingly focused on RCDs, due to their critical role in determining pathway specificity during AH degradation. Notably, although the differences between intradiol and extradiol RCDs initially seemed minor, subsequent studies revealed that these enzymes belong to two distinct and evolutionary unrelated protein classes (Vaillancourt et al. 2006). Thus far, all characterized intradiol dioxygenases belong to the same superfamily. In contrast, sequence and structural analyses have identified three families of unrelated extradiol dioxygenases (Edos): Type-I (e.g., catechol 2,3-dioxygenases) within the vicinal oxygen-chelate (VOC) superfamily, Type-II in the LigB superfamily (e.g., protocatechuate 4,5-dioxygenases), and Type-III enzymes belonging to the cupin superfamily (e.g., gentisate dioxygenases) (Vaillancourt et al. 2006). Additionally, a benzoquinol 1,2-dioxygenase identified in *Pseudomonas fluorescen*s ACB exhibits no significant sequence homology to known dioxygenases and has been proposed as the prototype of a novel Type-IV Edo (Pérez-Pantoja et al. 2010).

Type-III Edos are increasingly recognized for their role in the para-cleavage pathway, the third aerobic pathway for aromatic ring cleavage (Fig. 1). This pathway targets aromatic compounds with hydroxyl groups in the para-orientation (e.g., gentisates and hydroquinones) or those with a hydroxyl group adjacent to a carboxylate or amino group (e.g., salicylate, 2-aminophenol, and 1-hydroxy-2-naphthoate) (Ferraroni et al. 2013). An atypical type-III Edo is the gentisate 1,2-dioxygenase from *Pseudaminobacter salicylatoxidans* BN12. This enzyme shows broad substrate specificity, oxidizing gentisate and other monohydroxylated substrates (e.g., 1-hydroxy-2-naphthoate, substituted salicylates). These unique features have established type-III Edos as valuable models for exploring structure-function relationships within this family (Ferraroni et al. 2013; Subbotina et al. 2021).

### Anaerobic catabolic pathways for the degradation of MAHs

In anoxic environments, bacteria metabolize aromatic compounds by replacing O₂ with alternative electron acceptors, such as nitrate (NO₃⁻), sulfate (SO₄²⁻), carbon dioxide (CO₂), and ferric iron (Fe³⁺) (Pandolfo et al. 2023). Representative bacteria anaerobically degrading AHs include the denitrifiers *Thauera aromatica* and *Azoarcus evansii*, as well as the photoheterotroph *Rhodopseudomonas palustris* and the strict anaerobe *Geobacter metallireducens* (Porter and Young 2014). Like their aerobic counterparts, anaerobic microorganisms funnel AHs into central intermediates, often as Coenzyme-A (CoA) thioesters, either directly or following substrate activation. Most monoaromatic compounds are funnelled to the benzoyl-CoA central intermediate (Porter and Young 2014; Phale et al. 2020). However, other key intermediates, such as resorcinol, phloroglucinol, and hydroxyhydroquinone, have also been identified (Durante-Rodríguez et al. 2018; Boll et al. 2020).

**Fig. 2:**
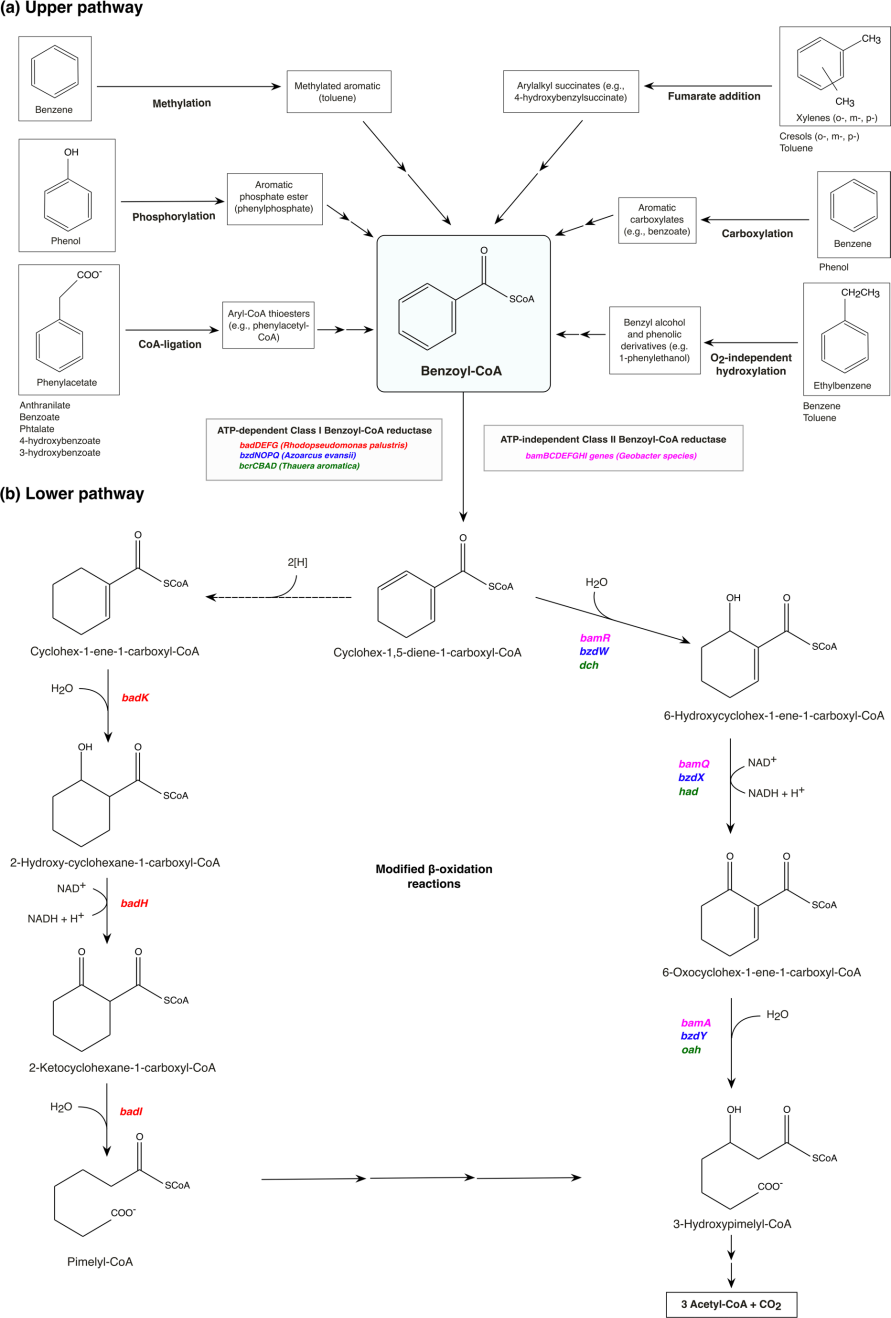
Anaerobic catabolism of monoaromatic hydrocarbons through the Benzoyl-CoA pathway. In the upper pathway **(a)**, diverse monoaromatic substrates are funneled into the common intermediate benzoyl-CoA (light-blue box) through distinct activation strategies including ATP-dependent CoA-ligation, phosphorylation, methylation, fumarate addition, carboxylation, and O_2_-independent hydroxylation. Boxes illustrate representative aromatic compounds activated by each mechanism; additional substrates listed below each box are likewise activated by the same mechanism but display distinct chemical structures. In the lower pathway **(b)**, aromatic ring reduction of benzoyl-CoA is carried out by either an ATP-dependent Class I benzoyl-CoA reductase encoded by clusters such as *badDEFG* in *R. palustris*, *bzdNOPQ* in *A. evansii*, or *bcrCBAD* in *T. aromatica*; or by an ATP-independent Class II reductase encoded by the *bamBCDEFGHI* gene cluster (*Geobacter* species). Next in the pathway, the reduced intermediate can follow two slightly different β-oxidation-like pathways leading to the formation of pimelyl-CoA in *R. palustris* and 3-hydroxypimelyl-CoA in *Thauera*,* Azoarcus* and *Geobacter* species. Genes involved in each step are color-coded according to the corresponding model organism: red (*R. palustris*), blue (*A. evansii*), green (*T. aromatica*) and pink (*Geobacter* species). Enzymatic steps include a cyclohexa-1,5-dienecarbonyl-CoA hydratase (encoded by *bamR/bzdW/dch*), 6-hydroxycyclohex-1-ene-1-carbonyl-CoA dehydrogenase (*bamQ/bzdX/had*), and 6-oxocyclohex-1-ene-carbonyl-CoA hydrolase (*bamA/bzdY/oah*). In *R. palustris*, the downstream reactions are carried out by a cyclohex-1-ene-1-carboxyl-CoA hydratase, a 2-hydroxycyclohexanecarboxyl-CoA dehydrogenase, and a 2-ketocyclohexanecarboxyl-CoA hydrolase, encoded by *badK*,* badH*, and *badI*, respectively. Dashed arrows indicate hypothetical enzymatic reactions. Sequential arrows indicate successive enzymatic transformations

Several biochemical strategies have been described for the activation of the aromatic ring, including fumarate addition, phosphorylation, O₂-independent hydroxylation, methylation, direct carboxylation, and ATP-dependent CoA ligation (Ladino-Orjuela et al. 2016; Hernández-Ospina et al. 2024). In contrast, the downstream degradation pathway for the benzoyl-CoA appears to be more conserved (Fig. 2).

Benzoate provides a model substrate for studying the benzoyl-CoA pathway (Boll et al. 2020). In the upper pathway, benzoate is activated to benzoyl-CoA by benzoate-CoA ligase (BCL). This enzyme belongs to the aryl-CoA ligases/synthetases (ACL) family, a broad group of enzymes that catalyze the ATP-dependent thioesterification of various monoaromatic acids. Genes encoding BCL have been identified in various anaerobes and are named differently across studied organisms, such as *R. palustris* (*badA*), *T. aromatica* (*bclA*), *Azoarcus* spp. (*bzdA*), and *G. metallireducens* (*bamY*) (Carmona et al. 2009). Interestingly, other ACLs have also been shown to recognize benzoate as a substrate for activation, including 4-chlorobenzoate-CoA ligase, 2-aminobenzoate-CoA ligase, and 4-hydroxybenzoate-CoA ligase (Arnold et al. 2021).

In the lower pathway, benzoyl-CoA undergoes reductive dearomatization catalyzed by benzoyl-CoA reductase (BCR), yielding cyclohexa-1,5-diene-1-carboxyl-CoA (dienoyl-CoA). The complete degradation of this compound then proceeds via β-oxidation-like reactions to yield acetyl-CoA and CO₂ (Boll et al. 2020). Facultative and obligate anaerobes use three enzymatic steps to convert dienoyl-CoA to hydroxypimelyl-CoA. The exception is *R. palustris*, where benzoyl-CoA is reduced twice to cyclohex-1-ene-1-carboxyl-CoA and further metabolized to pimelyl-CoA through a modified β-oxidation pathway (Schmid et al. 2015; Durante-Rodríguez et al. 2018). At present, it remains unclear whether additional variants of the benzoyl-CoA degradation pathway exist in other bacteria.

While analogous BCLs mediate benzoate activation, phylogenetic studies reveal that the subsequent dearomatization step is catalyzed by two distinct classes of BCRs, suggesting independent evolutionary origins in nature. Class I BCRs catalyze ATP-dependent, irreversible reactions and were long thought to occur exclusively in facultative anaerobes. However, recent genomic and biochemical studies revealed the presence of *Azoarcus*-type class I BCRs (*bzdNOPQ*) in the strictly anaerobic archaeon *Ferroglobus placidus*, thereby extending their distribution beyond facultative bacteria. In contrast, class II BCRs are ATP-independent, reversible enzymes widely distributed among strictly anaerobic bacteria, such as *Geobacter* and *Syntrophus* (Schmid et al. 2015; Tiedt et al. 2018; Boll et al. 2020). Despite their mechanistic differences, both classes share extreme sensitivity to oxygen, which accounts for their absence in aerobic organisms. The genes encoding BCR subunits are arranged into clusters, such as *bcrCBAD* (*T. aromatica*) and *bamBCDEFGHI* (*G. metallireducens*) (Boll et al. 2014), ensuring coordinate expression of the multimeric enzyme complex.

### Hybrid catabolic pathways for the degradation of AHs

**Fig. 3:**
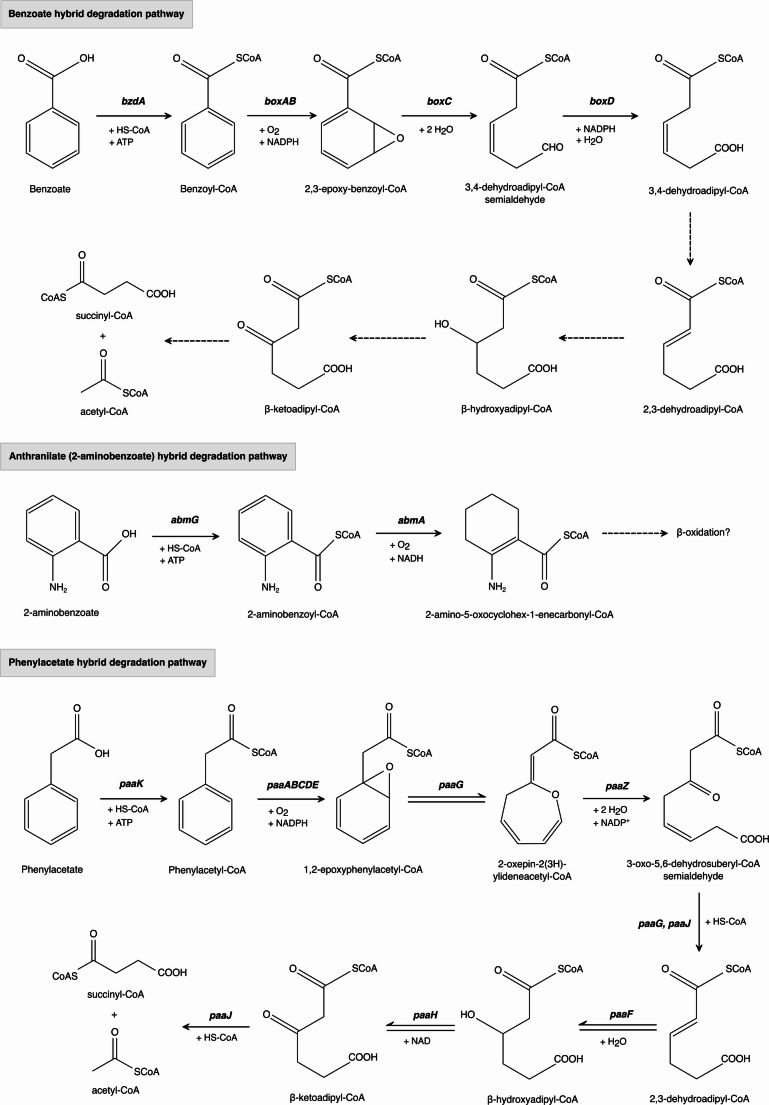
Aerobic hybrid pathways for benzoate, 2-aminobenzoate (anthranilate) and phenylacetate degradation in ***Azoarcus*** sp**.** Aromatic compounds are initially activated to benzoyl-CoA through ATP-dependent Aryl-CoA ligase, a mechanism typical of anaerobic pathways. However, the subsequent ring dearomatization proceeds through an oxygen-dependent epoxidation followed by hydrolytic ring opening, distinct from both classic aerobic (dioxygenase-based) and strictly anaerobic (reductive) pathways. Genes: *bzdA*, Benzoate-CoA ligase; *boxAB*, benzoyl-CoA 2,3-epoxidase; *boxC*, benzoyl-CoA-dihydrodiol lyase; *boxD*, 3,4-dehydroadipyl-CoA semialdehyde dehydrogenase; *abmG*, 2-aminobenzoate-CoA ligase; *abmA*, 2-aminobenzoyl-CoA monooxygenase; *paaK*, phenylacetate-CoA ligase; *paaABCDE*, ring-1,2-phenylacetyl-CoA epoxidase; *paaG*, ring-1,2-epoxy-phenylacetate-CoA isomerase; *paaZ*, oxepin-CoA hydrolase/3-oxo-5,6-dehydrosuberyl-CoA semialdehyde dehydrogenase; *paaJ*, 3-oxoadipyl-CoA/3-oxo-5,6-dehydrosuberyl-CoA thiolase; *paaF*, 2,3-dehydroadipyl-CoA hydratase; *paaH*, 3-hydroxyadipyl-CoA dehydrogenase. Dashed arrows indicate hypothetical enzymatic reactions. Question marks denote an entirely speculative step

A third degradation strategy for mineralizing benzoate and other aromatic compounds, such as phenylacetate (Phe) and anthranilate, has been experimentally identified in a limited number of bacterial species (Díaz et al. 2013; Arora 2015). These novel pathways, termed *hybrid pathways*, involve an initial activation of the aromatic ring through CoA thioesterification, followed by epoxidation, and an oxygen-independent hydrolytic ring cleavage (Fig. 3) (Fuchs et al. 2011). The *box pathway* from *A. evansii* was the first hybrid strategy identified (Gescher et al. 2002; Godínez-Pérez et al. 2024). This pathway begins with the activation of benzoate to benzoyl-CoA by a BCL that differs from its anaerobic counterpart in both molecular mass and chromosomal gene location (Arnold et al. 2021). Benzoyl-CoA is then subjected to oxygen-dependent epoxidation by the multicomponent monooxygenase BoxAB, followed by hydrolytic ring cleavage that bypasses the need for dioxygenases typically required in aerobic degradation. Subsequent steps involve oxidation, isomerization, and hydroxylation reactions that lead to the yet-undetected but likely intermediate β-ketoadipyl-CoA.

In silico analysis revealed that *box* genes are present in many α- and β-proteobacteria and some δ-proteobacteria (Fuchs et al. 2011). Furthermore, it has been reported that several strains harbor both the classical anaerobic and hybrid pathways for benzoate degradation, which are expressed depending on oxygen availability during growth (Valderrama et al. 2012). The utilization of CoA thioesters as initial intermediates has been suggested to offer advantages to microaerophilic and facultative anaerobes by allowing them to unify the uptake mechanism of aromatic compounds (Kawaguchi et al. 2006). This is particularly beneficial under fluctuating oxygen conditions, as a decrease below a critical oxygen threshold would permit a rapid and energetically efficient transition between aerobic and anaerobic degradation routes.

Recently, there has been increasing interest in characterizing ACL enzymes, as they are essential for generating the central benzoyl-CoA intermediate. However, biochemical data for most ACL enzymes and associated pathways remain limited. To date, only a few ACLs have been purified from bacteria capable of growing aerobically or anaerobically on aromatic substrates (Arnold et al. 2021). These enzymes exhibit broad substrate specificity, recognizing and activating benzoate and its structural analogs such as phenylacetate, 2-, 3-, and 4-fluorobenzoate, 2-aminobenzoate, and 3- and 4-hydroxybenzoate, into their corresponding CoA-thioesters. Structural and kinetic characterizations have so far been conducted only for the CoA ligases of benzoate, phenylacetate, anthranilate, and 4-chlorobenzoate, providing valuable insights into their catalytic mechanisms and the key residues determining substrate specificity (Bains and Boulanger 2007; Erb et al. 2008; Gulick et al. 2004).

### Microbial strategies for PAHs degradation

**Fig. 4:**
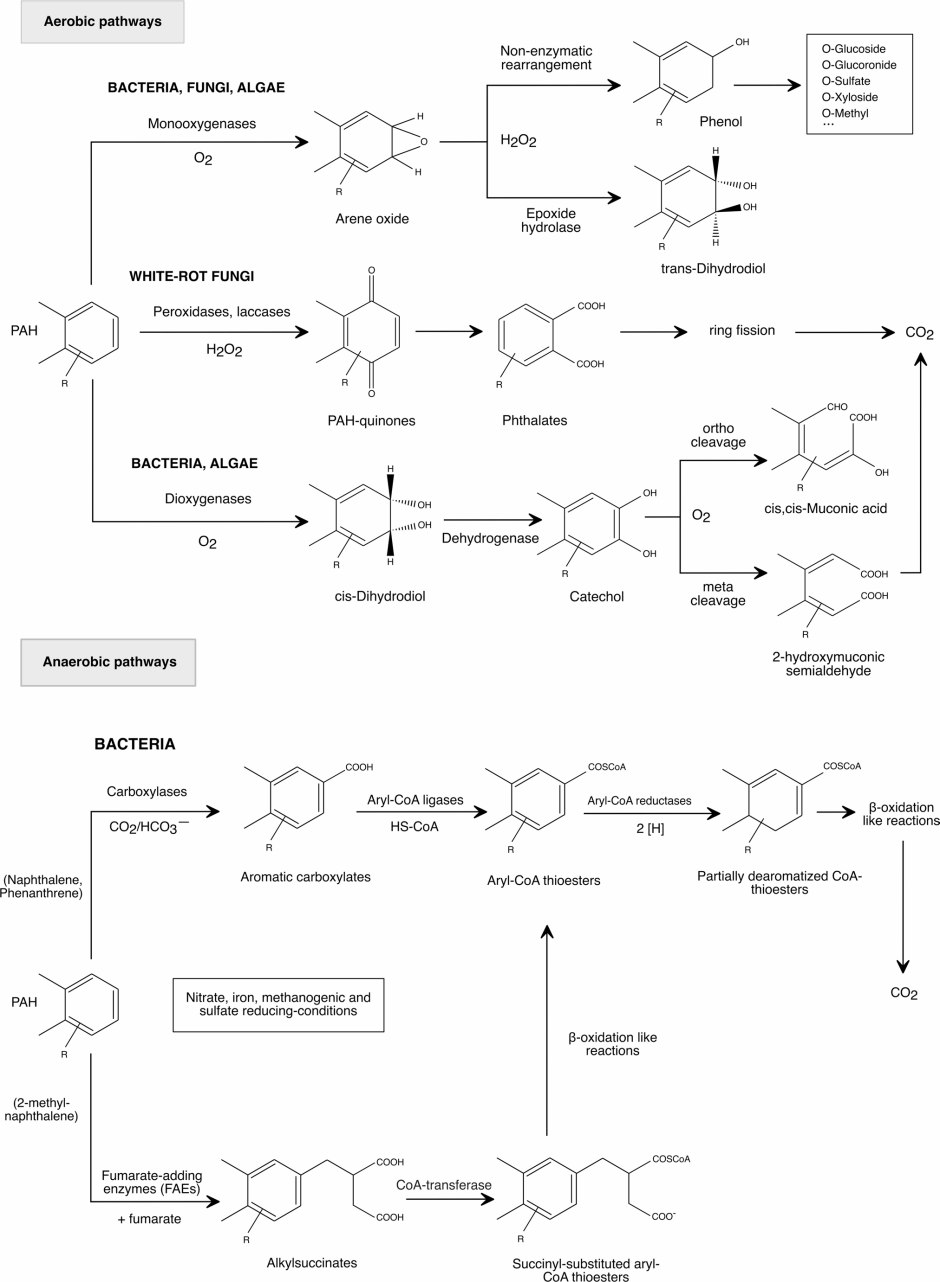
Degradation of PAHs by microorganisms. In aerobic microorganisms (bacteria, fungi, algae), PAHs are initially oxidized by monooxygenases or dioxygenases, yielding arene oxides or cis-dihydrodiols, which are further transformed to catechols or phenols. White-rot fungi employ peroxidases and laccases, producing PAH-quinones and phthalates. These intermediates undergo subsequent transformations, including dehydrogenation and ring-cleavage reactions (ortho or meta), ultimately leading to mineralization into CO₂. In anaerobic bacteria, LMW-PAHs are activated by carboxylation, CoA ligation, or fumarate addition. The resulting intermediates are reduced by aryl-CoA reductases and further metabolized through β-oxidation-like reactions

The degradation rate of PAHs is primarily influenced by physicochemical factors, including the number of aromatic rings, temperature, and water solubility. LMW PAHs, being more volatile and water-soluble, are more readily degraded by bacteria, whereas HMW PAHs pose a greater challenge due to their low solubility and limited bioavailability. Additionally, bacteria might lack the specific enzymes needed to initiate the breakdown of these complex molecules. Therefore, the complete mineralization of PAHs in natural environments often depends on the synergistic activity of diverse microorganisms. In microbial consortia, fungi and algae can initiate partial oxidation reactions. These include peroxidase- or laccase-mediated transformations into quinones or phthalates, which increase the bioavailability of otherwise recalcitrant PAHs (Hoque et al. 2023; Mou et al. 2023). These intermediates can subsequently be metabolized by bacteria through aerobic or anaerobic routes, ultimately leading to ring fission and mineralization to CO₂. Figure 4 summarizes the primary microbial pathways involved in the degradation of PAHs under both aerobic and anaerobic conditions, as well as the auxiliary oxidative contributions of fungi and algae that enhance the bioavailability of PAHs.

Similar to monoaromatic compounds, the aerobic degradation of PAHs in bacteria typically begins with a hydroxylation step catalyzed by ring-hydroxylating dioxygenases (RHDs), producing cis-dihydrodiols that are then oxidized to catechols. However, some *Mycobacterium* strains can mineralize PAHs through cytochrome P450 monooxygenase activity, yielding trans-dihydrodiols as intermediates (Cao et al. 2022). Aerobic degradation of PAHs is well documented for model substrates such as naphthalene, anthracene, and phenanthrene. Among the best-characterized systems is the *nah* operon in *P. putida* G7 (plasmid NAH7), which encodes enzymes for the conversion of naphthalene into salicylate (upper pathway) and subsequently into pyruvate and acetaldehyde (lower pathway) (Haritash and Kaushik 2009).

Whereas fungi and algae can effectively oxidize PAHs aerobically, bacteria display a remarkable ability to degrade PAHs anaerobically in many natural and polluted sites. Nevertheless, the underlying mechanisms of anaerobic PAHs degradation remain poorly characterized. To date, anaerobic degradation has been partially elucidated for LMW-PAHs such as naphthalene, 2-methylnaphthalene, and phenanthrene. In these pathways, strictly and facultative anaerobes initiate aromatic ring activation using some biochemical strategies described for monoaromatics. Under sulfate-reducing conditions, 2-methyl-naphthalene is activated by fumarate addition, similar to anaerobic toluene degradation. The unsubstituted PAHs naphthalene and phenanthrene are activated through carboxylation to 2-naphthoate/2-phenanthroate by UbiD-like carboxylases, followed by CoA-thioesters formation through ACLs (Samak et al. 2025). Subsequent dearomatization proceeds via ATP-independent reductions catalyzed by a novel class of Aryl-CoA reductases (ACRs).

### Biotechnological potential of AHs degradation

Beyond expanding ecological and biochemical knowledge, elucidating microbial pathways for AHs degradation provides new opportunities for biotechnological applications.

Field studies have demonstrated enhanced in situ bioremediation through bioaugmentation and stimulation of native bacterial degraders. *Pseudomonas putida* bioaugmentation enhanced treatment of phenolic landfill leachate in sequencing batch reactors (Michalska et al. 2020), and immobilized *Pseudomonas* spp. removed BTEX from contaminated groundwater in a fibrous-bed bioreactor (Shim and Yang 2002). Anaerobic degraders expand their applicability to oxygen-limited sites (e.g., sediments, aquifers), while consortia can outperform single strains (Forján et al. 2020; Hoque et al. 2023).

Hydrocarbon-degrading enzymes have also found applications in industrial biocatalysis. For instance, styrene monooxygenase has been employed for the enantioselective epoxidation of styrene to produce (S)-styrene oxide at pilot scale, a valuable precursor for pharmaceuticals and fragrances (Panke et al. 2002). Toluene dioxygenase has been engineered to enhance its activity for the dihydroxylation of bulky and ester-functionalized aromatics (Osifalujo et al. 2022). Currently, oxygenases involved in central pathways, such as catechol 1,2-dioxygenase, are now viewed less for their ecological role and more as strategic biocatalysts enabling renewable routes to industrial monomers like adipic acid (Kruyer et al. 2020). Synthetic biology has further expanded the use of aromatic-degrading enzymes by integrating them into heterologous pathways to drive the sustainable production of value-added compounds (Curran et al. 2013; Han et al. 2015).

## Omics-driven insights into AH degradation pathways

**Fig. 5:**
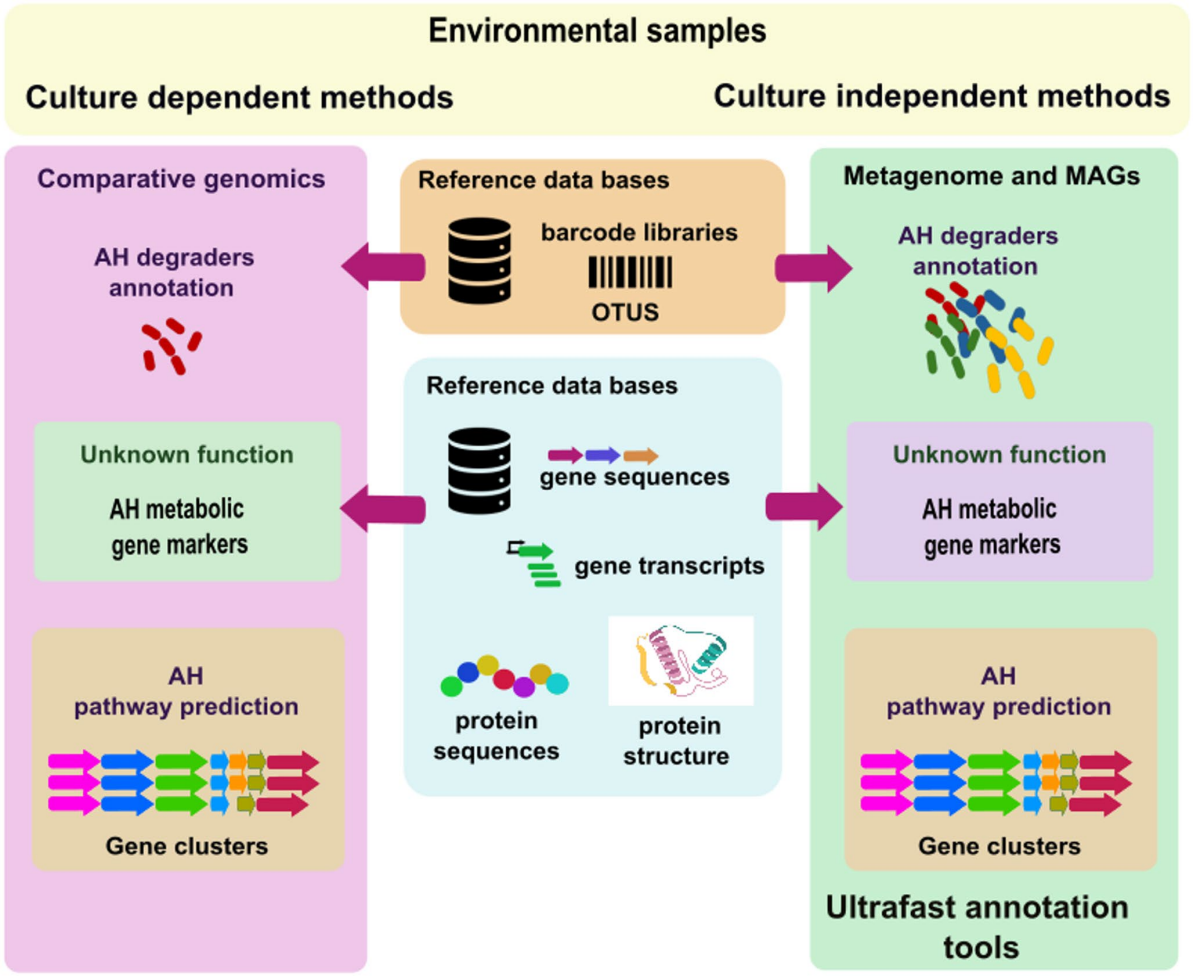
Strategies for exploring aromatic hydrocarbon degradation from environmental samples. Culture-dependent approaches and culture-independent methods, enable degrader annotation, identification of metabolic gene markers, and pathway prediction from gene clusters. Both rely on reference databases that integrate barcode libraries, gene and protein sequences, transcripts, and protein structures

In recent years, the integration of omics technologies and bioinformatic tools has greatly advanced the study of microbial catabolism. A key driver of this paradigm shift has been the advancement of sequencing technologies. Sequencing strategies have evolved from early chemical methods and manual gel-based techniques to automated fluorescent methods, culminating in modern high-throughput platforms, including next-generation sequencing (NGS) and long-read technologies. These approaches now facilitate the rapid and large-scale characterization of microbial genomes, including those of uncultured or low-abundance organisms. At the same time, multi-omics data provide complementary insights into gene expression, protein function, and metabolic activity. Several databases and predictive tools/resources currently assist the processing, integration, and interpretation of these large-scale datasets (Fig. 5).

Today, the increased availability of sequence genomes, combined with core bioinformatic tools, such as BLAST for rapid sequence alignment (Altschup et al. 1990), MMseqs2 for sensitive clustering of homologs (Steinegger and Söding 2017), MEME for de novo motif discovery (Bailey and Elkan 1994), and HMMER for profile-based homology detection (Potter et al. 2018), enables the functional prediction of previously uncharacterized enzymes involved in AHs degradation across diverse taxa. To enhance the accuracy and scalability of these predictions, protein-family models built from multiple sequence alignments and profile Hidden Markov Models (HMMs) are curated in specialized databases such as Pfam (Mistry et al., 2021). These resources serve as components for annotation pipelines like RAST/RASTtk (Brettin et al. 2015) and eggNOG-mapper-v2 (Cantalapiedra et al., 2021), which automate functional annotation across genomes or metagenomes. Such annotations are further enriched by cross-referencing with biochemical and enzymatic databases, including KEGG (Kanehisa et al., 2016), MetaCyc (Krieger et al. 2004), BRENDA (Chang et al. 2021), UniProt (Bateman et al. 2023), PDB (Berman et al. 2002), and PubChem (Kim et al. 2021), which provide curated insights into enzyme function, metabolic reactions, and chemical structures.

**Fig. 6:**
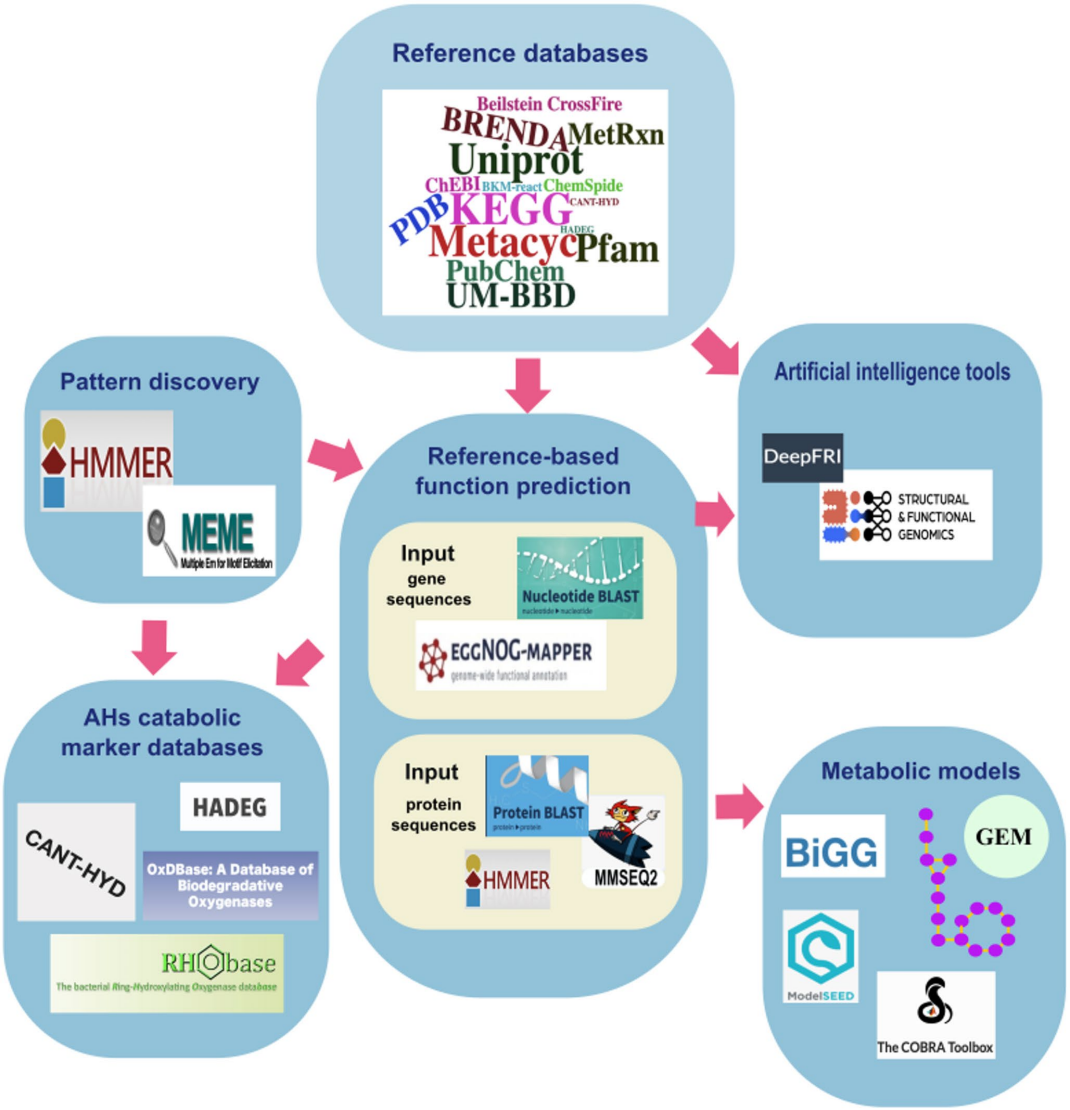
Bioinformatic tools and resources for the functional prediction of genes, enzymes, and pathways involved in AHs degradation. Reference databases (e.g., KEGG, UniProt, Pfam, MetaCyc) provide essential information on genes, proteins, and metabolic pathways. Pattern discovery tools such as HMMER and MEME are used to detect conserved domains and sequence motifs, while specialized AH catabolic marker databases (CANT-HYD, HADEG, OxDBase, RHObase) compile curated data on genes and enzymes directly involved in AH degradation. Gene and protein sequences can be analyzed through reference-based function prediction tools (BLAST, eggNOG-mapper, MMSEQ2, HMMER), with annotations further improved using artificial intelligence approaches (e.g., DeepFRI). Finally, predicted functions and pathways can be incorporated into genome-scale metabolic models (BiGG, ModelSEED, GEMs, COBRA Toolbox) to reconstruct metabolic networks and evaluate the ecological or biotechnological roles of AH degraders

Together, these resources have consolidated biochemical, enzymatic, and structural information, enabling accurate function assignment, genome-wide identification of candidate AHs–degrading enzymes, and the reconstruction of complete catabolic pathways from genomic and metagenomic data (Fig. 6).

### Comparative genomics

Comparative genomics is the systematic comparison of biological information derived from whole-genome sequences (de Crécy-Lagard and Hanson 2013). This approach supports gene annotation, metabolic potential prediction, and the reconstruction of metabolic and regulatory networks associated with AHs degradation (Suvorova and Gelfand 2019; Khot et al. 2022). Such efforts have led to the discovery of genes encoding novel AHs-degrading enzymes. In *Desulfatiglans* TRIP_1, Kraiselburd et al. (2019) combined MicroScope, KEGG, MetaCyc, CheckM, and homology searches with transcriptomic and proteomic data to propose a phenanthroate-CoA ligase and UbiD-like carboxylases involved in phenanthrene degradation. Building on these predictions, Kaplieva-Dudek et al. (2024) later purified and characterized the 2-phenanthroate-CoA ligase, providing biochemical validation of its activity.

Broader comparative genomics studies have shown the extensive genetic potential for aromatic catabolism across diverse bacteria. For example, a comparative genomics study in *Cupriavidus necator* JMP134 enabled the prediction of numerous catabolic pathways through genome sequencing, in silico annotation, gene-context analysis, and phylogenetic reconstruction. These predictions were experimentally validated, as the strain was shown to grow on 60 aromatic substrates, metabolized through the β-ketoadipate, gentisate, homogentisate, phenylacetyl-CoA, and benzoyl-CoA pathways (Pérez-Pantoja et al. 2008).

Genomic analyses are now extending this approach beyond single-organism genomes. A survey of 80 *Burkholderiales* genomes, using BLASTP searches and phylogenetic reconstruction of 48 oxygenase markers, revealed nearly all central ring-cleavage pathways (Pérez-Pantoja et al. 2012). More recently, genome sequencing and annotation (RASTtk, DRAM) of 22 bacterial strains from contaminated soils identified hundreds of genes for the degradation of benzoate, gentisate, homogentisate, protocatechuate, BTEX, and LMW-PAHs (Hossain et al. 2024).

### Metagenomics and metagenome-assembled genomes

Metagenomics enables the study of uncultured microbes directly from environmental DNA, revealing both community composition and the metabolic potential of complex microbial consortia involved in AHs biodegradation. Functional annotation of metagenomes typically relies on optimized comparative-genomics tools (Fig. 5). Ultrafast homology searches against reference protein databases such as UniProt and NCBI RefSeq (O’Leary et al. 2016) are commonly performed with DIAMOND (Buchfink et al. 2021), while orthology-based tools like eggNOG-mapper v2 are now standard for large-scale functional inference in environmental datasets. Additionally, the recovery of metagenome-assembled genomes (MAGs) yields near-complete or draft genomes reconstructed from community datasets via assembly and binning (Yang et al. 2021). Through MAGs, we can better understand microbial diversity, reconstruct metabolism, and study AH degradation in natural and polluted environments, from deep-sea sediments to polluted soils.

Case studies underscore the shift from tools to discoveries. Stable isotope probing (SIP) coupled with shotgun metagenomics identified uncultured *Rhodocyclaceae* as dominant phenanthrene degraders in contaminated soils, enabling the cloning of novel RHDs with distinct substrate specificities. MAGs recovered from a refinery wastewater treatment plant under nitrogen limitation showed that *Bradyrhizobiaceae* encode nitrogen fixation pathways and at least 14 oxygenases for AH utilization (Tikariha and Purohit 2019). More recently, Artificial Intelligence (AI)-guided metagenome mining, which combined HMM profiles, structural modeling, and functional validation, uncovered sequence-divergent dioxygenases/peroxidases that were missed by homology searches (Nagy et al. 2024).

As a natural extension of these findings, specialized resources such as HADEG, CANT-HYD, RHObase, and OxDBase have emerged to support hydrocarbon degradation research. HADEG compiles experimentally validated genes involved in aerobic degradation, serving as a reference framework for reliable annotation (Chakraborty et al. 2014; Rojas-Vargas et al. 2023). CANT-HYD provides a set of 37 HMMs designed for the identification and annotation of marker genes involved in anaerobic and aerobic degradation, making it suitable for large-scale genome and metagenome annotation (Khot et al. 2022). In contrast, RHObase and OxDBase focus on oxygenase enzymes, providing sequence, structural, and functional information, along with datasets that can be directly used to perform BLAST searches for functional prediction (Arora et al. 2009; Chakraborty et al. 2014). While these resources represent valuable efforts, the number of bioinformatic tools specifically designed for AHs degradation research remains very limited, and most of them are focused on aerobic rather than anaerobic processes. This highlights the need for resources that address the full range of microbial catabolic strategies.

### Multi-omics integration

While genomics and metagenomics reveal the genetic potential of microbial communities, they provide only a static inventory of possible functions. Capturing the active processes that determine AHs degradation requires additional omics layers such as transcriptomics, proteomics, and metabolomics. These approaches enable the transition from prediction to direct observation of regulation, expression, and metabolic outcomes in situ.

Transcriptomics describes the dynamic gene expression under specific environmental conditions. For example, RNA-Seq studies have shown the induction of genes encoding RHDs and peripheral catabolic enzymes when bacteria are exposed to AHs, thereby validating predictions derived from genomic data (Luo et al. 2014; Peng et al. 2020; Zampolli et al. 2020). Moreover, transcriptomic profiling allows the discovery of novel regulators and transcriptional networks that fine-tune catabolic pathways in response to substrate availability or stress (Martinez-Varela et al. 2023). This approach has also been highlighted in the context of synthetic biology, where transcriptomics is considered a key step to characterize hydrocarbon-responsive regulatory systems and to repurpose them as biosensors (Moratti et al. 2022).

Proteomics complements transcriptomics approaches by giving direct information about enzymes rather than RNA. Techniques such as Liquid Chromatography–Tandem Mass Spectrometry (LC–MS/MS), have been employed to quantify the expression of degrading-enzymes such as oxygenases, CoA ligases, and reductases. For example, Heintz et al. (2009) used differential membrane proteomics to reveal the first ATP-independent Class II BCR in *Geobacter metallireducens*. Similarly, proteomic profiling of *Rhodococcus* sp. TFB indicated induction of enzymes specific to phthalate and naphthalene metabolism, enabling the reconstruction of a complete phthalate degradation pathway (Tomás-Gallardo et al. 2006). A proteogenomic study in *Burkholderia* sp. K24 confirmed the coexistence of multiple independent pathways for aniline, benzoate, and toluene degradation (Lee et al. 2016). More recently, proteomic surveys have emphasized their value in detecting post-translational modifications, enzyme isoforms, and unexpected metabolic interactions, thus providing a way to complement transcriptomic predictions (Kim et al. 2009).

Metabolomics adds the downstream dimension by profiling small molecules and pathway intermediates. Through techniques such as Gas Chromatography–Mass Spectrometry **(**GC–MS) or Nuclear Magnetic Resonance (NMR), metabolomics allows detection of diagnostic intermediates. For instance, GC–MS has been employed to identify catechol and cis, cis-muconate during phenol degradation, thereby validating the ortho-cleavage pathway in yeast cells (Filipowicz et al. 2020), and to detect deuterated phenol as the initial intermediate in benzene degradation by halophilic *Arhodomonas* sp., confirming hydroxylation as the entry step (Dalvi et al. 2012). Complementary targeted metabolomics of CoA-thioesters using LC–MS/MS has also uncovered activated intermediates such as halobenzoyl-CoA and benzoyl-CoA in anaerobic degraders, providing chemical evidence for pathway variants (Kuntze et al. 2011; Cakić et al. 2021).

When integrated, these omics approaches create a multi-layered systems view of AHs degradation. As such, multi-omics integration bridges the gap between genetic potential and realized function.

## Emerging approaches

AI and machine learning (ML) now enhance functional genomics by predicting enzyme functions and metabolic potential. DeepFRI (Gligorijević et al. 2021) infers function from sequence and structural features even in the absence of detectable homology. AntiSMASH and ML classifiers have predicted biosynthetic gene clusters across bacterial, fungal, and plant genomes within the ANL superfamily, which includes ACLs central to AHs degradation (Robinson et al. 2020). The ML-classified ANLs enabled phylogenetic reconstruction, suggesting ancient ANLs had active sites most similar to modern enzymes that use CoA-SH as the acceptor.

Genome-scale metabolic models (GEMs), via BiGG (Norsigian et al. 2020), ModelSEED (Seaver et al. 2021), MOST (Kelley et al. 2017), and the COBRA toolbox (Heirendt et al. 2019), integrate these predictions into networks, enabling in silico flux and pathway simulations. For example, the reconstruction of *Pseudomonas putida* KT2440 using COBRA enabled flux balance simulations that validated the catabolic pathway for toluene degradation (Nogales et al. 2008). Similarly, a curated GEM of *P. fluorescens* predicted catechol degradation through the β-ketoadipate pathway using RAST and ModelSEED (Huang and Lin 2020).

Despite their growing utility, both AI-based predictions and GEM reconstructions remain limited by annotation gaps, biased training datasets, and the lack of experimental validation. Addressing these challenges will require integrating curated multi-omics data, improving modeling algorithms, and incorporating biochemical evidence to fully exploit their potential in elucidating AHs degradation pathways.

## Limitations, future perspectives, and conclusions

Omics and bioinformatics have deepened our understanding of the degradation of AHs, revealing new enzymes, extending known pathways to overlooked taxa, and uncovering alternative routes. Genomics and metagenomics are now essential for probing niches where cultured and uncultured microbes work together to degrade and mineralize AHs.

Despite these advances, our biochemical understanding of key anaerobic steps remains incomplete. For example, the proposed anaerobic benzene carboxylase (UbiD/UbiX-type) is supported by omics evidence yet remains only partially resolved biochemically. Likewise, the activation of naphthalene via carboxylation in the sulfate-reducing culture N47 has been mechanistically outlined, but the corresponding enzyme is still being consolidated. In the case of phenanthrene, downstream steps involving 2-phenanthroate-CoA ligase and 2-phenanthroyl-CoA reductase have been defined, yet the identity and diversity of the initial activating-carboxylase remain to be fully elucidated. These gaps highlight the importance of integrating omics predictions with experimental validation to unravel the full enzymatic potential of environmental microbes.

Another key limitation in AHs research is the shortage of specialized databases and tools for predicting catabolic genes and enzymes. Existing resources and reference databases are mostly restricted to aerobic pathways, focusing on oxygenases and other well-characterized enzymes (e.g., OxDBase, RHObase). Consequently, key anaerobic enzymes (e.g., aryl-CoA ligases, carboxylases, aryl-CoA reductases) remain poorly annotated and are rarely incorporated into predictive workflows. In parallel, reference datasets are dominated by model organisms like *Pseudomonas* and *Escherichia coli*, leaving uncultivated lineages underrepresented. As a result, catabolic strategies encoded in MAGs or single-cell genomes are often missed, and annotations in complex microbiomes can be incomplete or biased.

Finally, an additional challenge is the poor integration of omics layers that provide convergent evidence of AHs degradation pathways. Metagenomics, transcriptomics, proteomics, and metabolomics offer complementary insights, but they are rarely unified into predictive frameworks. Likewise, AI approaches that move beyond homology are seldom connected to experimental omics or metabolic models, which limits their ability to predict the function of divergent or poorly characterized enzyme families.

Addressing these limitations will require coordinated efforts to expand specialized databases, improve predictive frameworks, and link computational models more closely with ecological conditions. Progress will depend on incorporating enzymes reconstructed from MAGs and single-cell genomes, extending taxonomic coverage, and enriching catabolic annotations. Integrating genome-scale metabolic models with ecological simulations that account for oxygen gradients, nutrient levels, and pollutant concentrations could further bridge the gap between laboratory predictions and field conditions. Such advances will enhance prediction accuracy, facilitate the discovery of key microbial degraders and enzymes, and ultimately unlock the full bioremediation potential of microbial communities.

## Data Availability

No datasets were generated or analysed during the current study.

## Abbreviations

AHs: Aromatic hydrocarbons
MAHs: Monocyclic aromatic hydrocarbons
PAHs: Polycyclic aromatic hydrocarbons
LMW PAHs: Low molecular weight PAHs
HMW PAHs: High molecular weight PAHs
TCA: Tricarboxylic acid
RCDs: Ring-cleaving dioxygenases
RHDs: Ring-hydroxylating dioxygenases
β-KAP: β-ketoadipate pathway
Edos: Extradiol dioxygenases
VOC: Vicinal oxygen-chelate
CoA: Coenzyme-A
BCL: Benzoate-CoA ligase
ACL: Aryl-CoA ligase/synthetase
BCR: Benzoyl-CoA reductase
Phe: Phenanthrene
HMMs: Hidden Markov Models
NGS: Next-generation sequencing
MAGs: Metagenome-assembled genomes
SIP: Stable isotope probing
GEMs: Genome-scale metabolic models
AI: Artificial Intelligence
ML: Machine learning
GC–MS: Gas Chromatography–Mass Spectrometry
NMR: Nuclear Magnetic Resonance
LC–MS/MS: Techniques such as Liquid Chromatography–Tandem Mass Spectrometry

## References

[CR1] Altschup SF, Gish W, Miller W, Myers EW, Lipman DJ (1990) Basic Local Alignment Search Tool 10.1016/S0022-2836(05)80360-2 2231712

[CR2] Arnold ME, Kaplieva-Dudek I, Heker I, Meckenstock RU (2021) Aryl coenzyme A ligases, a subfamily of the Adenylate-Forming enzyme superfamily. Appl Environ Microbiol. 10.1128/AEM 34260306 10.1128/AEM.00690-21 PMC8388817

[CR3] Arora PK (2015) Bacterial degradation of monocyclic aromatic amine. Front Microbiol 6. 10.3389/fmicb.2015.00820 10.3389/fmicb.2015.00820 PMC4539516 26347719

[CR4] Arora PK, Kumar M, Chauhan A, Raghava GP, Jain RK (2009) OxDBase: a database of oxygenases involved in biodegradation. BMC Res Notes. 10.1186/1756-0500-2-67 19405962 10.1186/1756-0500-2-67 PMC2683861

[CR5] Bailey TL, Elkan C (1994) Fitting a mixture model by expectation maximization to discover motifs in biopolymers. Proceedings of the International Conference on Intelligent Systems for Molecular Biology:28–36 7584402

[CR6] Bains J, Boulanger MJ (2007) Biochemical and structural characterization of the paralogous benzoate coa ligases from *Burkholderia xenovorans* LB400: defining the entry point into the novel benzoate oxidation (box) pathway. J Mol Biol 373(4):965–977. 10.1016/j.jmb.2007.08.008 17884091 10.1016/j.jmb.2007.08.008

[CR7] Bateman A, Martin MJ, Orchard S, Magrane M, Ahmad S, Alpi E et al (2023) UniProt: the universal protein knowledgebase in 2023. Nucleic Acids Res 51(D1):D523–D531. 10.1093/nar/gkac1052 36408920 10.1093/nar/gkac1052 PMC9825514

[CR8] Berman HM, Battistuz T, Bhat TN, Bluhm WF, Bourne PE, Burkhardt K, Feng Z, Gilliland GL, Iype L, Jain S, Fagan P, Marvin J, Padilla D, Ravichandran V, Schneider B, Thanki N, Weissig H, Westbrook JD, Zardecki C (2002) Biological crystallography the protein data bank. Acta Cryst 58:899–907 10.1107/s0907444902003451 12037327

[CR9] Boll M, Löffler C, Morris BEL, Kung JW (2014) Anaerobic degradation of homocyclic aromatic compounds via arylcarboxyl-coenzyme A esters: organisms, strategies and key enzymes. Environ Microbiol 16(3):612–627. 10.1111/1462-2920.12328 24238333 10.1111/1462-2920.12328

[CR10] Boll M, Estelmann S, Heider J (2020) Catabolic pathways and enzymes involved in the anaerobic degradation of monocyclic aromatic compounds. Anaerobic utilization of hydrocarbons, oils, and lipids. Springer International Publishing, pp 85–133

[CR11] Brettin T, Davis JJ, Disz T, Edwards RA, Gerdes S, Olsen GJ, Olson R, Overbeek R, Parrello B, Pusch GD, Shukla M, Thomason JA, Stevens R, Vonstein V, Wattam AR, Xia F (2015) RASTtk: a modular and extensible implementation of the RAST algorithm for building custom annotation pipelines and annotating batches of genomes. Sci Rep. 10.1038/srep08365 25666585 10.1038/srep08365 PMC4322359

[CR12] Buchan A, Neidle EL, Moran MA (2004) Diverse organization of genes of the β-ketoadipate pathway in members of the marine Roseobacter lineage. Appl Environ Microbiol 70(3):1658–1668. 10.1128/AEM.70.3.1658-1668.2004 15006791 10.1128/AEM.70.3.1658-1668.2004 PMC368412

[CR13] Buchfink B, Reuter K, Drost HG (2021) Sensitive protein alignments at tree-of-life scale using DIAMOND. Nat Methods 18(4):366–368. 10.1038/s41592-021-01101-x 33828273 10.1038/s41592-021-01101-x PMC8026399

[CR14] Cakić N, Kopke B, Rabus R, Wilkes H (2021) Suspect screening and targeted analysis of acyl coenzyme A thioesters in bacterial cultures using a high-resolution tribrid mass spectrometer. Anal Bioanal Chem 413:3599–3610. 10.1007/s00216-021-03318-3/Published 33881564 10.1007/s00216-021-03318-3 PMC8141488

[CR15] Cantalapiedra CP, Hern̗andez-Plaza A, Letunic I, Bork P, Huerta-Cepas J (2021) eggNOG-mapper v2: functional annotation, orthology assignments, and domain prediction at the metagenomic scale. Mol Biol Evol 38(12):5825–5829. 10.1093/molbev/msab293 10.1093/molbev/msab293 PMC8662613 34597405

[CR16] Cao Z, Yan W, Ding M, Yuan Y (2022) Construction of microbial consortia for microbial degradation of complex compounds. Front Bioeng Biotechnol. 10.3389/fbioe.2022.1051233 36561050 10.3389/fbioe.2022.1051233 PMC9763274

[CR17] Carmona M, Zamarro MT, Blázquez B, Durante-Rodríguez G, Juárez JF, Valderrama JA, Barragán MJL, García JL, Díaz E (2009) Anaerobic catabolism of aromatic compounds: a genetic and genomic view. Microbiol Mol Biol Rev 73(1):71–133. 10.1128/mmbr.00021-08 19258534 10.1128/MMBR.00021-08 PMC2650882

[CR18] Chakraborty J, Jana T, Saha S, Dutta TK (2014) Ring-hydroxylating oxygenase database: a database of bacterial aromatic ring-hydroxylating oxygenases in the management of bioremediation and biocatalysis of aromatic compounds. Environ Microbiol Rep 6(5):519–523. 10.1111/1758-2229.12182 25646545 10.1111/1758-2229.12182

[CR19] Chang A, Jeske L, Ulbrich S, Hofmann J, Koblitz J, Schomburg I, Neumann-Schaal M, Jahn D, Schomburg D (2021) Brenda, the ELIXIR core data resource in 2021: new developments and updates. Nucleic Acids Res 49(D1):D498–D508. 10.1093/nar/gkaa1025 33211880 10.1093/nar/gkaa1025 PMC7779020

[CR20] Curran KA, Leavitt JM, Karim AS, Alper HS (2013) Metabolic engineering of muconic acid production in *Saccharomyces cerevisiae*. Metab Eng 15(1):55–66. 10.1016/j.ymben.2012.10.003 23164574 10.1016/j.ymben.2012.10.003

[CR21] Dalvi S, Azetsu S, Patrauchan MA, Aktas DF, Fathepure BZ (2012) Proteogenomic elucidation of the initial steps in the benzene degradation pathway of a novel halophile, *Arhodomonas* sp. strain rozel, isolated from a hypersaline environment. Appl Environ Microbiol 78(20):7309–7316. 10.1128/AEM.01327-12 22885747 10.1128/AEM.01327-12 PMC3457091

[CR22] de Crécy-Lagard V, Hanson A (2013) Comparative genomics. Brenner’s encyclopedia of genetics: second edition. Elsevier Inc., pp 102–105

[CR23] Díaz E, Jiménez JI, Nogales J (2013) Aerobic degradation of aromatic compounds. Curr Opin Biotechnol 24:431–442 23122741 10.1016/j.copbio.2012.10.010

[CR24] Durante-Rodríguez G, Gómez-Álvarez H, Blázquez B, Fernández-Llamosas H, Martín-Moldes Z, Sanz D, Nogales J, Carmona M, Díaz E (2018) Chap. 13: anaerobic pathways for the catabolism of aromatic compounds. RSC energy and environment series. Royal Society of Chemistry, pp 333–390

[CR25] Erb TJ, Ismail W, Fuchs G (2008) Phenylacetate metabolism in thermophiles: characterization of phenylacetate-CoA ligase, the initial enzyme of the hybrid pathway in *Thermus thermophilus*. Curr Microbiol 57(1):27–32. 10.1007/s00284-008-9147-3 18414813 10.1007/s00284-008-9147-3

[CR26] Ferraroni M, Matera I, Bürger S, Reichert S, Steimer L, Scozzafava A, Stolz A, Briganti F (2013) The salicylate 1,2-dioxygenase as a model for a conventional gentisate 1,2-dioxygenase: crystal structures of the G106A mutant and its adducts with gentisate and salicylate. FEBS J 280(7):1643–1652. 10.1111/febs.12173 23384287 10.1111/febs.12173

[CR27] Filipowicz N, Momotko M, Boczkaj G, Cieśliński H (2020) Determination of phenol biodegradation pathways in three psychrotolerant yeasts, *Candida subhashii* A011, *Candida oregonensis* B021 and *Schizoblastosporion starkeyi-henricii* L012, isolated from Rucianka peatland. Enzyme Microb Technol. 10.1016/j.enzmictec.2020.109663 33051016 10.1016/j.enzmictec.2020.109663 PMC7474889

[CR28] Forján R, Lores I, Sierra C, Baragaño D, Gallego JLR, Peláez AI (2020) Bioaugmentation treatment of a PAH-polluted soil in a slurry bioreactor. Appl Sci. 10.3390/APP10082837

[CR29] Fuchs G, Boll M, Heider J (2011) Microbial degradation of aromatic compounds- from one strategy to four. Nat Rev Microbiol 9:803–816 21963803 10.1038/nrmicro2652

[CR30] Gescher J, Zaar A, Mohamed M, Schägger H, Fuchs G (2002) Genes coding for a new pathway of aerobic benzoate metabolism in *Azoarcus evansii*. J Bacteriol 184(22):6301–6315. 10.1128/JB.184.22.6301-6315.2002 12399500 10.1128/JB.184.22.6301-6315.2002 PMC151953

[CR31] Ghosal D, Ghosh S, Dutta TK, Ahn Y (2016) Corrigendum to Current state of knowledge in microbial degradation of polycyclic aromatic hydrocarbons (PAHs): A review [Front. Microbiol. 2016, 7:1369]. 10.3389/fmicb.2016.01369. Front Microbiol 7 10.3389/fmicb.2016.01369 PMC5006600 27630626

[CR32] Gligorijević V, Renfrew PD, Kosciolek T, Leman JK, Berenberg D, Vatanen T, Chandler C, Taylor BC, Fisk IM, Vlamakis H, Xavier RJ, Knight R, Cho K, Bonneau R (2021) Structure-based protein function prediction using graph convolutional networks. Nat Commun. 10.1038/s41467-021-23303-9 34039967 10.1038/s41467-021-23303-9 PMC8155034

[CR33] Godínez-Pérez CM, Loza A, Hurtado JM, Gutiérrez-Ríos RM (2024 ) The benzoyl-CoA pathway serves as a genomic marker to identify the oxygen requirements in the degradation of aromatic hydrocarbons. Front Microbiol 14. 10.3389/fmicb.2023.1308626 10.3389/fmicb.2023.1308626 PMC10803450 38264488

[CR34] Gulick AM, Lu X, Dunaway-Mariano D (2004) Crystal structure of 4-chlorobenzoate:CoA ligase/synthetase in the unliganded and aryl substrate-bound statest. Biochemistry 43(27):8670–8679. 10.1021/bi049384m 15236575 10.1021/bi049384m

[CR35] Han L, Liu P, Sun J, Wu Y, Zhang Y, Chen W, Lin J, Wang Q, Ma Y (2015) Engineering catechol 1, 2-dioxygenase by design for improving the performance of the *Escherichia coli* synthetic pathway in *Escherichia coli*. Sci Rep. 10.1038/srep13435 26306712 10.1038/srep13435 PMC4549619

[CR36] Haritash AK, Kaushik CP (2009) Biodegradation aspects of polycyclic aromatic hydrocarbons (PAHs): a review. J Hazard Mater 169:1–15 19442441 10.1016/j.jhazmat.2009.03.137

[CR37] Heintz D, Gallien S, Wischgoll S, Ullmann AK, Schaeffer C, Kretzschmar AK, Van Dorsselaer A, Boll M (2009) Differential membrane proteome analysis reveals novel proteins involved in the degradation of aromatic compounds in *Geobacter metallireducens*. Mol Cell Proteomics 8(9):2159–2169. 10.1074/mcp.M900061-MCP200 19497847 10.1074/mcp.M900061-MCP200 PMC2742446

[CR38] Heirendt L, Arreckx S, Pfau T, Mendoza SN, Richelle A, Heinken A, Haraldsdóttir HS, Wachowiak J, Keating SM, Vlasov V, Magnusdóttir S, Yu Ng C, Preciat G, Žagare A, Chan SHJ, Aurich MK, Clancy CM, Modamio J, Sauls JT, Noronha A, Bordbar A, Cousins B, El Assal DC, Valcarcel LV, Fleming RMT (2019) Creation and analysis of biochemical constraint-based models using the COBRA toolbox v.3.0. Nat Protoc 2(3):639–738. 10.1038/nprot.2007.99 10.1038/s41596-018-0098-2 PMC6635304 30787451

[CR39] Hernández-Ospina DA, Osorio-González CS, Miri S, Kaur Brar S (2024) New perspectives on the anaerobic degradation of BTEX: mechanisms, pathways, and intermediates. Chemosphere. 10.1016/j.chemosphere.2024.142490 38821131 10.1016/j.chemosphere.2024.142490

[CR40] Hoque MZ, Sankaran S, Anand D, Musa MM, Nzila A, Guerriero G, Siddiqui KS, Ahmad I (2023) Enhanced biodegradation of phenanthrene and anthracene using a microalgal-bacterial consortium. Front Microbiol. 10.3389/fmicb.2023.1227210 37771703 10.3389/fmicb.2023.1227210 PMC10525690

[CR41] Hossain MS, Iken B, Iyer R (2024) Whole genome analysis of 26 bacterial strains reveals aromatic and hydrocarbon degrading enzymes from diverse environmental soil samples. Sci Rep. 10.1038/s41598-024-78564-3 39730399 10.1038/s41598-024-78564-3 PMC11680993

[CR42] Huang X, Lin YH (2020) Reconstruction and analysis of a three-compartment genome-scale metabolic model for *Pseudomonas fluorescens*. Biotechnol Appl Biochem 67(1):133–139. 10.1002/bab.1852 31721286 10.1002/bab.1852

[CR43] Kanehisa M, Sato Y, Kawashima M, Furumichi M, Tanabe M (2016) KEGG as a reference resource for gene and protein annotation. Nucleic Acids Res 44(D1):D457–D462. 10.1093/nar/gkv1070 10.1093/nar/gkv1070 PMC4702792 26476454

[CR44] Kaplieva-Dudek I, Samak NA, Bormann J, Kaschani F, Kaiser M, Meckenstock RU (2024) Characterization of 2-phenanthroate:CoA ligase from the sulfate-reducing, phenanthrene-degrading enrichment culture TRIP. Appl Environ Microbiol. 10.1128/aem.01296-24 39248461 10.1128/aem.01296-24 PMC11497795

[CR45] Kawaguchi K, Shinoda Y, Yurimoto H, Sakai Y, Kato N (2006) Purification and characterization of benzoate-CoA ligase from *Magnetospirillum* sp. strain TS-6 capable of aerobic and anaerobic degradation of aromatic compounds. FEMS Microbiol Lett 257(2):208–213. 10.1111/j.1574-6968.2006.00165.x 16553855 10.1111/j.1574-6968.2006.00165.x

[CR46] Kelley JJ, Maor S, Kim MK, Lane A, Lun DS (2017) Most-visualization: software for producing automated textbook-style maps of genome-scale metabolic networks. Bioinformatics 33(16):2596–2597. 10.1093/bioinformatics/btx240 28430868 10.1093/bioinformatics/btx240

[CR47] Khot V, Zorz J, Gittins DA, Chakraborty A, Bell E, Bautista MA, Paquette AJ, Hawley AK, Novotnik B, Hubert CRJ, Strous M, Bhatnagar S (2022) CANT-HYD: a curated database of phylogeny-derived hidden Markov models for annotation of marker genes involved in hydrocarbon degradation. Front Microbiol. 10.3389/fmicb.2021.764058 35069469 10.3389/fmicb.2021.764058 PMC8767102

[CR48] Kim SJ, Kweon O, Cerniglia CE (2009) Proteomic applications to elucidate bacterial aromatic hydrocarbon metabolic pathways. Curr Opin Microbiol 12:301–309 19414279 10.1016/j.mib.2009.03.006

[CR49] Kim S, Chen J, Cheng T, Gindulyte A, He J, He S, Li Q, Shoemaker BA, Thiessen PA, Yu B, Zaslavsky L, Zhang J, Bolton EE (2021) PubChem in 2021: new data content and improved web interfaces. Nucleic Acids Res 49(D1):D1388–D1395. 10.1093/nar/gkaa971 33151290 10.1093/nar/gkaa971 PMC7778930

[CR50] Kraiselburd I, Brüls T, Heilmann G, Kaschani F, Kaiser M, Meckenstock RU (2019) Metabolic reconstruction of the genome of candidate *Desulfatiglans* TRIP_1 and identification of key candidate enzymes for anaerobic phenanthrene degradation. Environ Microbiol 21(4):1267–1286. 10.1111/1462-2920.14527 30680888 10.1111/1462-2920.14527 PMC6849830

[CR51] Krieger CJ, Zhang P, Mueller LA, Wang A, Paley S, Arnaud M, Pick J, Rhee SY, Karp PD (2004) MetaCyc: A multiorganism database of metabolic pathways and enzymes. Nucleic Acids Res 32. 10.1093/nar/gkh100. (DATABASE ISS.) 10.1093/nar/gkh100 PMC308834 14681452

[CR52] Kruyer NS, Wauldron N, Bommarius AS, Peralta-Yahya P (2020) Fully biological production of adipic acid analogs from branched catechols. Sci Rep. 10.1038/s41598-020-70158-z 32770001 10.1038/s41598-020-70158-z PMC7414886

[CR53] Kuntze K, Kiefer P, Baumann S, Seifert J, von Bergen M, Vorholt JA, Boll M (2011) Enzymes involved in the anaerobic degradation of meta-substituted halobenzoates. Mol Microbiol 82(3):758–769. 10.1111/j.1365-2958.2011.07856.x 22010634 10.1111/j.1365-2958.2011.07856.x

[CR54] Ladino-Orjuela G, Gomes E, da Silva R, Salt C, Parsons JR (2016) Metabolic pathways for degradation of aromatic hydrocarbons by bacteria. Reviews of environmental contamination and toxicology. Springer, New York LLC, pp 105–121 10.1007/978-3-319-23573-8_5 26613990

[CR55] Lee SY, Kim GH, Yun SH, Choi CW, Yi YS, Kim J, Chung YH, Park EC, Kim S, Il (2016) Proteogenomic characterization of monocyclic aromatic hydrocarbon degradation pathways in the aniline-degrading bacterium *Burkholderia* sp. K24. PLoS ONE 11(4). 10.1371/journal.pone.0154233 10.1371/journal.pone.0154233 PMC4849787 27124467

[CR56] Luo F, Gitiafroz R, Devine CE, Gong Y, Hug LA, Raskin L, Edwards EA (2014) Metatranscriptome of an anaerobic benzene-degrading, nitrate-reducing enrichment culture reveals involvement of carboxylation in benzene ring activation. Appl Environ Microbiol 80(14):4095–4107. 10.1128/AEM.00717-14 24795366 10.1128/AEM.00717-14 PMC4068673

[CR57] Martinez-Varela A, Casas G, Berrojalbiz N, Lundin D, Piña B, Dachs J, Vila-Costa M (2023) Metatranscriptomic responses and microbial degradation of background polycyclic aromatic hydrocarbons in the coastal mediterranean and Antarctica. Environ Sci Pollut Res Int 30(57):119988–119999. 10.1007/s11356-023-30650-1 37934408 10.1007/s11356-023-30650-1 PMC10697874

[CR58] Martins M, Costa PM, Ferreira AM, Costa MH (2013) Comparative DNA damage and oxidative effects of carcinogenic and non-carcinogenic sediment-bound PAHs in the gills of a bivalve. Aquat Toxicol 142:85–95. 10.1016/j.aquatox.2013.07.019 23969285 10.1016/j.aquatox.2013.07.019

[CR59] Michalska J, Piński A, Zur J, Mrozik A (2020) Analysis of the bioaugmentation potential of *Pseudomonas Putida* OR45a and *Pseudomonas Putida* KB3 in the sequencing batch reactors fed with the phenolic landfill leachate. Water (Switzerland) 12(3). 10.3390/w12030906

[CR60] Mistry J, Chuguransky S, Williams L, Qureshi M, Salazar GA, Sonnhammer ELL, Tosatto SCE, Paladin L, Raj S, Richardson LJ, Finn RD, Bateman A (2021) Pfam: the protein families database in 2021. Nucleic Acids Res 49(D1):D412–D419. 10.1093/nar/gkaa913 10.1093/nar/gkaa913 PMC7779014 33125078

[CR61] Moratti CF, Scott C, Coleman NV (2022) Synthetic biology approaches to hydrocarbon biosensors: a review. Front Bioeng Biotechnol. 10.3389/fbioe.2021.804234 35083206 10.3389/fbioe.2021.804234 PMC8784404

[CR62] Mou B, Gong G, Wu S (2023) Biodegradation mechanisms of polycyclic aromatic hydrocarbons: combination of instrumental analysis and theoretical calculation. Chemosphere. 10.1016/j.chemosphere.2023.140017 37657699 10.1016/j.chemosphere.2023.140017

[CR63] Nagy KK, Takács K, Németh I, Varga B, Grolmusz V, Molnár M, Vértessy BG (2024) Novel enzymes for biodegradation of polycyclic aromatic hydrocarbons identified by metagenomics and functional analysis in short-term soil microcosm experiments. Sci Rep. 10.1038/s41598-024-61566-6 38773163 10.1038/s41598-024-61566-6 PMC11109138

[CR64] Nogales J, Palsson B, Thiele I (2008) A genome-scale metabolic reconstruction of *Pseudomonas Putida* KT2440: iJN746 as a cell factory. BMC Syst Biol. 10.1186/1752-0509-2-79 18793442 10.1186/1752-0509-2-79 PMC2569920

[CR65] Norsigian CJ, Pusarla N, McConn JL, Yurkovich JT, Dräger A, Palsson BO, King Z (2020) Bigg models 2020: multi-strain genome-scale models and expansion across the phylogenetic tree. Nucleic Acids Res 48(D1):D402–D406. 10.1093/nar/gkz1054 31696234 10.1093/nar/gkz1054 PMC7145653

[CR66] O’Leary NA, Wright MW, Brister JR, Ciufo S, Haddad D, McVeigh R, Rajput B, Robbertse B, Smith-White B, Ako-Adjei D, Astashyn A, Badretdin A, Bao Y, Blinkova O, Brover V, Chetvernin V, Choi J, Cox E, Ermolaeva O, Farrell CM, Goldfarb T, Gupta T, Haft D, Hatcher E, Hlavina W, Joardar VS, Kodali VK, Li W, Maglott D, Masterson P, McGarvey KM, Murphy MR, O’Neill K, Pujar S, Rangwala SH, Rausch D, Riddick LD, Schoch C, Shkeda A, Storz SS, Sun H, Thibaud-Nissen F, Tolstoy I, Tully RE, Vatsan AR, Wallin C, Webb D, Wu W, Landrum MJ, Kimchi A, Tatusova T, DiCuccio M, Kitts P, Murphy TD, Pruitt KD (2016) Reference sequence (RefSeq) database at NCBI: current status, taxonomic expansion, and functional annotation. Nucleic Acids Res 44(D1):D733–D745. 10.1093/nar/gkv1189 26553804 10.1093/nar/gkv1189 PMC4702849

[CR67] Osifalujo EA, Preston-Herrera C, Betts P, Satterwhite LR, Froese JT (2022) Improving toluene dioxygenase activity for Ester-Functionalized substrates through enzyme engineering. ChemistrySelect 7(11). 10.1002/slct.202200753

[CR68] Pandolfo E, Barra Caracciolo A, Rolando L (2023) Recent advances in bacterial degradation of hydrocarbons. Water. 10.3390/w15020375

[CR69] Panke S, Held M, Wubbolts MG, Witholt B, Schmid A (2002) Pilot-scale production of (S)-styrene oxide from styrene by recombinant *Escherichia coli* synthesizing styrene monooxygenase. Biotechnol Bioeng 80(1):33–41. 10.1002/bit.10346 12209784 10.1002/bit.10346

[CR70] Peng T, Kan J, Hu J, Hu Z (2020) Genes and novel sRNAs involved in PAHs degradation in marine bacteria *Rhodococcus* sp. P14 revealed by the genome and transcriptome analysis. 3 Biotech. 10.1007/s13205-020-2133-6 32206489 10.1007/s13205-020-2133-6 PMC7044393

[CR71] Pérez-Pantoja D, De La Iglesia R, Pieper DH, González B (2008) Metabolic reconstruction of aromatic compounds degradation from the genome of the amazing pollutant-degrading bacterium *Cupriavidus necator* JMP134. FEMS Microbiol Rev 32:736–794 18691224 10.1111/j.1574-6976.2008.00122.x

[CR72] Pérez-Pantoja D, Donoso R, Junca H, González B, Pieper DH (2010) Phylogenomics of aerobic bacterial degradation of aromatics. Handbook of hydrocarbon and lipid microbiology. Springer, Berlin Heidelberg, pp 1355–1397

[CR73] Pérez-Pantoja D, Donoso R, Agulló L, Córdova M, Seeger M, Pieper DH, González B (2012) Genomic analysis of the potential for aromatic compounds biodegradation in burkholderiales. Environ Microbiol 14:1091–1117 22026719 10.1111/j.1462-2920.2011.02613.x

[CR74] Phale PS, Malhotra H, Shah BA (2020) Degradation strategies and associated regulatory mechanisms/features for aromatic compound metabolism in bacteria. Advances in applied microbiology. Academic Press Inc., pp 1–65 10.1016/bs.aambs.2020.02.002 32762865

[CR75] Porter AW, Young LY (2014) Benzoyl-CoA, a universal biomarker for anaerobic degradation of aromatic compounds. Advances in applied microbiology. Academic Press Inc., pp 167–203 10.1016/B978-0-12-800260-5.00005-X 24767428

[CR76] Potter SC, Luciani A, Eddy SR, Park Y, Lopez R, Finn RD (2018) HMMER web server: 2018 update. Nucleic Acids Res 46(W1):W200–W204. 10.1093/nar/gky448 29905871 10.1093/nar/gky448 PMC6030962

[CR77] Robinson SL, Terlouw BR, Smith MD, Pidot SJ, Stinear TP, Medema MH, Wackett LP (2020) Global analysis of adenylate-forming enzymes reveals b-lactone biosynthesis pathway in pathogenic *nocardia*. J Biol Chem 295(44):14826–14839. 10.1074/jbc.RA120.013528 32826316 10.1074/jbc.RA120.013528 PMC7606675

[CR78] Rojas-Vargas J, Castelán-Sánchez HG, Pardo-López L (2023) HADEG: a curated hydrocarbon aerobic degradation enzymes and genes database. Comput Biol Chem. 10.1016/j.compbiolchem.2023.107966 37778093 10.1016/j.compbiolchem.2023.107966

[CR79] Samak NA, Götz F, Adjir K, Schaller T, Häßler M, Schmitz OJ, Fax J, Haberhauer G, Surmeneva A, Meckenstock RU (2025) Characterization of 2-phenanthroyl-CoA reductase, an ATP-independent type III aryl-CoA reductase involved in anaerobic phenanthrene degradation. Appl Environ Microbiol. 10.1128/aem.00166-25 40243319 10.1128/aem.00166-25 PMC12093976

[CR80] Schmid G, René SB, Boll M (2015) Enzymes of the benzoyl-coenzyme A degradation pathway in the hyperthermophilic archaeon Ferroglobus placidus. Environ Microbiol 17(9):3289–3300. 10.1111/1462-2920.12785 25630364 10.1111/1462-2920.12785

[CR81] Seaver SMD, Liu F, Zhang Q, Jeffryes J, Faria JP, Edirisinghe JN, Mundy M, Chia N, Noor E, Beber ME, Best AA, DeJongh M, Kimbrel JA, D’haeseleer P, McCorkle SR, Bolton JR, Pearson E, Canon S, Wood-Charlson EM, Cottingham RW, Arkin AP, Henry CS (2021) The modelseed biochemistry database for the integration of metabolic annotations and the reconstruction, comparison and analysis of metabolic models for plants, fungi and microbes. Nucleic Acids Res 49(D1):D575–D588. 10.1093/nar/gkaa746 32986834 10.1093/nar/gkaa746 PMC7778927

[CR82] Seo JS, Keum YS, Li QX (2009) Bacterial degradation of aromatic compounds. Int J Environ Res Public Health 6:278–309 19440284 10.3390/ijerph6010278 PMC2672333

[CR83] Shim H, Yang ST (2002) BTEX removal from contaminated groundwater by a co-culture of *Pseudomonas putida* and *Pseudomonas fluorescens* immobilized in a continuous fibrous-bed bioreactor. J Chem Technol Biotechnol 77(12):1308–1315. 10.1002/jctb.711

[CR84] Shrivastava R, Phale PS (2013) Biodegradation of mono-aromatic compounds by bacteria. In: Microorganisms in Environmental Management: Microbes and Environment. Springer Netherlands, pp 451–476

[CR85] Steinegger M, Söding J (2017) MMseqs2 enables sensitive protein sequence searching for the analysis of massive data sets. Nat Biotechnol 35:1026–1028 29035372 10.1038/nbt.3988

[CR86] Subbotina NM, Chernykh AM, Taranov AI, Shebanova AD, Moiseeva OV, Ferraroni M, Kolomytseva MP (2021) Gentisate 1,2-dioxygenase from the gram-positive bacteria *Rhodococcus opacus* 1CP: identical active sites vs. different substrate selectivities. Biochimie 180:90–103. 10.1016/j.biochi.2020.10.016 33122105 10.1016/j.biochi.2020.10.016

[CR87] Suvorova IA, Gelfand MS (2019) Comparative genomic analysis of the regulation of aromatic metabolism in Betaproteobacteria. Front Microbiol. 10.3389/fmicb.2019.00642 30984152 10.3389/fmicb.2019.00642 PMC6449761

[CR88] Tiedt O, Fuchs J, Eisenreich W, Boll M (2018) A catalytically versatile benzoyl-CoA reductase, key enzyme in the degradation of methyl- and halobenzoates in denitrifying bacteria. J Biol Chem 293(26):10264–10274. 10.1074/jbc.RA118.003329 29769313 10.1074/jbc.RA118.003329 PMC6028946

[CR89] Tikariha H, Purohit HJ (2019) Assembling a genome for novel nitrogen-fixing bacteria with capabilities for utilization of aromatic hydrocarbons. Genomics 111(6):1824–1830. 10.1016/j.ygeno.2018.12.005 30552976 10.1016/j.ygeno.2018.12.005

[CR90] Tomás-Gallardo L, Canosa I, Santero E, Camafeita E, Calvo E, López JA, Floriano B (2006) Proteomic and transcriptional characterization of aromatic degradation pathways in *Rhodoccocus* sp. strain TFB. Proteomics 6(Suppl 1):S119. 10.1002/pmic.200500422 16544280 10.1002/pmic.200500422

[CR91] Vaillancourt F, Bolin J, Eltis L (2006) The ins and outs of ring-cleaving dioxygenases. Crit Rev Biochem Mol Biol 41:241–267 16849108 10.1080/10409230600817422

[CR92] Valderrama JA, Durante-Rodríguez G, Blázquez B, García JL, Carmona M, Díaz E (2012) Bacterial degradation of benzoate: cross-regulation between aerobic and anaerobic pathways. J Biol Chem 287(13):10494–10508. 10.1074/jbc.M111.309005 22303008 10.1074/jbc.M111.309005 PMC3322966

[CR93] Wells T, Ragauskas AJ (2012) Biotechnological opportunities with the β-ketoadipate pathway. Trends Biotechnol 30:627–637 23122644 10.1016/j.tibtech.2012.09.008

[CR94] Yang C, Chowdhury D, Zhang Z, Cheung WK, Lu A, Bian Z, Zhang L (2021) A review of computational tools for generating metagenome-assembled genomes from metagenomic sequencing data. Comput Struct Biotechnol J 19:6301–6314 34900140 10.1016/j.csbj.2021.11.028 PMC8640167

[CR95] Zampolli J, Di Canito A, Cappelletti M, Collina E, Lasagni M, Di Gennaro P (2020) Biodegradation of naphthenic acids: identification of *Rhodococcus opacus* R7 genes as molecular markers for environmental monitoring and their application in slurry microcosms. Appl Microbiol Biotechnol 104(6):2675–2689. 10.1007/s00253-020-10378-5 31993702 10.1007/s00253-020-10378-5

